# Modeling Spinal Cord Injury in a Dish with Hyperosmotic Stress: Population-Specific Effects and the Modulatory Role of Mesenchymal Stromal Cell Secretome

**DOI:** 10.3390/ijms26073298

**Published:** 2025-04-02

**Authors:** Jonas Campos, Ana T. Palha, Luís S. Fernandes, Jorge R. Cibrão, Tiffany S. Pinho, Sofia C. Serra, Nuno A. Silva, Adina T. Michael-Titus, António J. Salgado

**Affiliations:** 1 Life and Health Sciences Research Institute (ICVS), School of Medicine, Campus de Gualtar, University of Minho, 4710-057 Braga, Portugal; d9533@alunos.uminho.pt (J.C.); pg49203@alunos.uminho.pt (A.T.P.); pg49124@alunos.uminho.pt (L.S.F.); d8803@alunos.uminho.pt (J.R.C.); id8267@alunos.uminho.pt (T.S.P.); sofiaserra@med.uminho.pt (S.C.S.); nunosilva@med.uminho.pt (N.A.S.); 2 ICVS/3B’s—PT Government Associate Laboratory, 4805-017 Guimaraes, Portugal; 3 Centre for Neuroscience, Surgery and Trauma, The Blizard Institute, Barts and The London School of Medicine and Dentistry, Queen Mary University of London, London E1 2AT, UK; a.t.michael-titus@qmul.ac.uk

**Keywords:** spinal cord injury, osmotic shock, sorbitol, stem cells, MSCs, secretome, in vitro model

## Abstract

Innovations in spinal cord injury (SCI) models are crucial for developing effective therapies. This study introduces a novel in vitro SCI model using cultures of primary mixed spinal cord cells from rat pups, featuring key spinal cord cell types. This model offers distinct advantages in terms of feasibility, reproducibility, and cost-effectiveness, requiring only basic cell culture equipment. Following hyperosmotic stress via sorbitol treatment, the model recapitulated SCI pathophysiological hallmarks, with a 65% reduction in cell viability and gradual cell death over 48 h, making it ideal for evaluating neuroprotective agents. Notably, the human adipose tissue stem cell (hASC) secretome provided significant protection: it preserved metabolic viability, reduced β amyloid precursor protein (β-APP) expression in surviving neurons, and modulated the shift in the astrocytic morphotype. A transcriptomic profile of the effect of the hASC secretome treatment showed significant functional enrichments related to cell proliferation and cycle progression pathways. In addition to supporting the use of the hASC secretome as a therapy for SCI, this study is the first to use sorbitol as a hyperosmolar stressor to recapitulate key aspects of SCI pathophysiology. Thereby, this model can be used as a promising platform for evaluating therapeutic agents targeting neuroprotection and neuroregeneration, offering outputs related to cell death, neuronal stress, and protection, as well as induction of glial reactivity.

## 1. Introduction

A spinal cord injury (SCI) frequently results in an interruption of function below the injured area [1]. This condition, mainly attributed to traffic accidents, sports, and falls, has devastating consequences due to its high disability rate, morbidity, and economic burden [1,2,3]. The mechanical trauma leads first to the primary injury, where vascular disruption in the neural parenchyma damages the grey matter, with subsequent damages to the white matter [4,5]. This is followed by a complex cascade of neurodegenerative events, known as the secondary injury, categorized in acute, sub-acute, and chronic phases. The initial phases of the secondary injury mechanisms initiate after ischemic and hemorrhagic damage to the spinal cord parenchyma, eliciting membrane lipid peroxidation that leads to ionic imbalances and excitotoxicity [6,7,8]. These, in turn, promote massive cell death, generating an inflammatory environment that is perpetuated by several waves of infiltrative peripheral immune cells [9,10]. The chronic phase is marked by prolonged demyelination of axonal tracts and the establishment of a glial scar composed of several types of cells [11]. Secondary injury unfolds over days to weeks or even months after the initial injury [10]. The major challenge in managing the combination of massive cell death, complex inflammatory processes, and the growth inhibitory environment, as well as the successful induction of regenerative processes, are the primary causes of therapeutic failure [8,12].

Over the last few decades, there have been advances in in vitro models developed for studying the complex mechanisms behind SCI, as well as serving as platforms for therapeutic testing [13]. The existing cell culture paradigms for the in vitro study of SCI pathology suffer from two issues. The first has to do with the limited translational potential of simple two-dimensional models composed of one type of cell or cell line, despite being easy to implement and affordable. The second has to do with the relative difficulty of implementation and cost-inefficiency of complex three-dimensional systems and organoids [14,15]. Primary cell culture models are obtained from dissociated spinal cord tissues. They can vary from a culture enriched in a specific cell type to a mixed cell culture that contains the major neuronal and glial cells of the spinal cord [14,16]. Using mixed-population primary cultures can help address the inherent limitations of other complex in vitro models (such as organotypic preparations and organoids), being less time-consuming and offering better reproducibility [17]. In addition, developing a SCI “in a dish” model would allow a more comprehensive understanding of the multi-cellular interactions after injury, as well as the specific effects of therapeutic agents. The success and predictive validity of the injury induction itself will depend on the recapitulation of specific mechanisms of the SCI microenvironment that are present during secondary injury events [14,17,18]. This study had the objective to find a solution to these problems, by combining two innovative cell culture approaches. Firstly, we took advantage of well-known rat primary neuronal cell cultures to develop and validate a method to isolate spinal cord cells, to generate a mixed cell culture composed of the major spinal cord cell populations. Secondly, we established a novel SCI “in a dish” system to model hyperosmolar stress, as one of the first pathophysiological mechanisms involved in the secondary cascade following an SCI. In fact, it is known that there are significant osmotic changes in the spinal cord parenchyma right after injury, resulting from disruptions in the fluid and ion balance, with the additional accumulation of cellular damage from affected tissues [19,20]. To accomplish that, we used the known osmolyte sorbitol, which has been successfully employed in models of hyperosmolar perturbations [21,22,23,24]. It has been demonstrated that sorbitol administration for 1 h induces cellular stress mechanisms in primary mixed glial cultures, cortico-hippocampal brain slices, and induced pluripotent stem cell (iPSC)-derived motor neurons, which culminates in oxidative stress and DNA damage that ultimately lead to cell cycle arrest [21,25,26,27]. In this context, it is expected that hyperosmolar stress will provoke both direct and indirect cellular stress mechanisms that mimic key secondary injury events present in SCI.

Notably, this work is the first to use sorbitol as a tool to induce hyperosmolar stress as an in vitro SCI model, offering a promising platform for evaluating therapeutic agents targeting neuroprotection and neuroregeneration. As a proof-of-concept, this model was applied as a testing platform to the study of the neuroprotective impact of the human adipose tissue stem cell (hASC)-derived secretome, which has been emerging as a potential therapeutic approach for SCI [28,29,30].

## 2. Results

### 2.1. Development of a Mixed-Cell Spinal Cord Culture System

The complex pathophysiology of SCI generates a demand for the establishment of robust pre-clinical models that are able to generate relevant mechanistic insight and serve as a viable screening platform for novel therapeutic agents [31]. However, a majority of the in vitro models used to study SCI rely on cultures containing only one cell type, with a considerable bias toward neuronal-only cell cultures [14]. In this study, a novel hypertonic stress injury paradigm was established in a cell system that contained the major cell types from the spinal cord (neurons, astrocytes, microglia, and oligodendrocytes). Supplementary Figure S1 shows the detailed protocol for isolation of spinal cord cells through hydraulic extrusion. This protocol yields approximately one million cells on average per each post-natal day-five rat pup and facilitates the extraction of meninges-free spinal cord, a major hurdle in traditional dissection methods. Additionally, the protocol is considerably faster than traditional laminectomy-based protocols, as reported previously [32].

### 2.2. Characterization of Cell Adhesion and Growth

**Figure 1:**
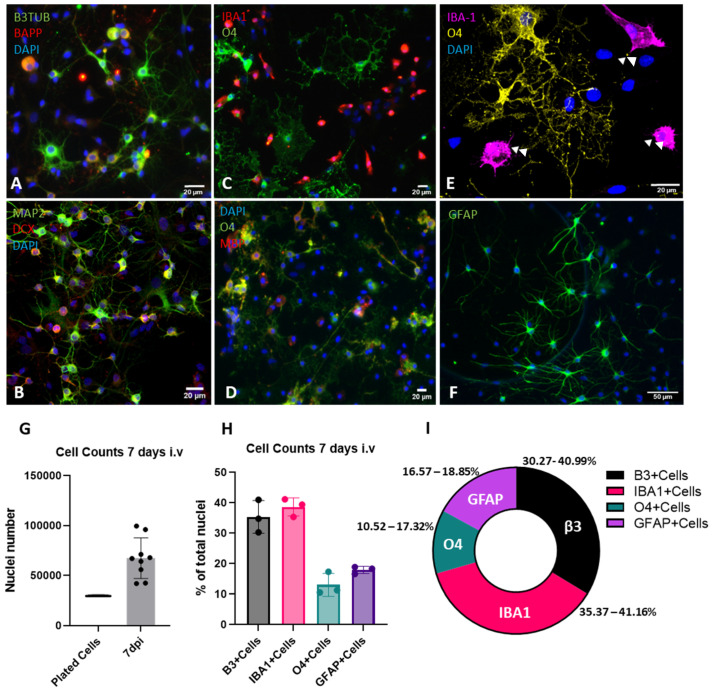
Immunocytochemical characterization of mixed spinal cord culture cell populations. (**A**–**F**) Lineage-specific marker expression patterns at 7 days post-plating. (**A**,**B**) Expression of neuronal markers. (**C**–**E**) Expression of microglia and oligodendroglial markers. (**F**) Expression of astrocyte marker. (**G**) Quantification of cellular density 7 days post-plating. (**H**) Quantification of lineage-specific markers expressed as a percentage of total cell nuclei based on the DAPI signal. Data are shown as median ± IQR for (**G**), mean ± SD for (**H**), and range for (**I**). Individual data points represent quantification of 3 individual assays. The Mann–Whitney test was used for (**G**). White arrow-heads point to microglial cells engulfing oligodendrocyte processes.

Given the complex interplay of multiple cell populations involved in the pathophysiological response to SCI, having knowledge of the cellular composition within cell cultures used in the SCI field is paramount. Therefore, immunofluorescence experiments using lineage-specific markers were conducted to characterize the cellular composition of the established cultures. Firstly, to understand the survival and growth dynamics of the plated cells, qualitative bright-field observations throughout the establishment of the cultures were carried out, and an imaging pipeline to quantify adhered cells at seven days in vitro was developed (Figure 1 and Supplementary Figure S2). This spatially unbiased cell number assessment was done based on whole-well images of 4′,6-diamidino-2-phenylindole DAPI-stained cell nuclei, followed by intensity-based threshold segmentation and watershed division of cell clusters. Bright-field observations showed the presence of developing cell types of all major lineages at 72 h post-plating, which matured at 7 days of culture (Supplementary Figure S2A,B).

### 2.3. Characterization of Cellular Populations

By using immunofluorescence techniques, the expression of markers of neuronal and glial cell populations was characterized, as well as cell densities based on DAPI image analysis (Figure 1A–I). Quantification results of cellular densities demonstrated that in 7 days, cell culture numbers roughly doubled, with the number of plated cells reaching an average of 67,000 cells/well in 96-well plates (Figure 1G), suggesting the fitness of the cultures and suitability for experimental manipulations. In Figure 1A–F, image panels show the different assessed markers. Immunofluorescence combination approaches showed that around 40% of neuronal cells constitutively expressed the beta-amyloid precursor protein (β-APP) during culture establishment, which is a marker related to cell stress adaptations (Figure 1A). This shows that β-APP expression dynamics could be used to assess pathological stress responses in neuronal cells within these cultures [33]. Also, expression of other neuronal markers, such as the newborn neuron marker doublecortin and the mature neuronal marker microtubule-associated protein 2 (MAP2) protein, was also present, demonstrating that ongoing neuronal maturation is a feature of this cell culture system (Figure 1A,B). This enables the use of such system to assess therapies focused on inducing neuronal differentiation and maturation, as well as axonal-specific phenotypes, such as fragmentation or regrowth. Cells committed to the oligodendroglial lineage were identified based on expression of the pre-myelinating marker O4 and myelination marker myelin basic protein (MBP) (Figure 1C–E). Microglial cells were identified by ionized calcium-binding adaptor molecule 1 (IBA-1) staining and were revealed to have a relative ameboid morphology (Figure 1C,E). Accordingly, this microglial morphotype is commonly found in spinal cord cultures [34]. When assessed by double-immunolabeling, microglial cells were seen to be in close association with oligodendrocytes and, in some instances, were observed to be phagocytosing myelin debris (Figure 1E), as pointed out by white arrows. The astroglial cell population was probed with the glial fibrilary acidic protein (GFAP) marker and appeared segregated as two distinct populations based on their morphotype (Figure 1F). These different morphologies are classically associated with their presence deriving from the grey or white matter; however, the understanding of the functional differences between fibrous or protoplasmic astrocytes is only beginning to emerge [35,36,37]. The quantification of the percentage of total cells expressing lineage-specific markers is presented in Figure 1H,I. The data demonstrating the percentage ranges of marker expression showed that neuronal and microglial cells were the prevalent cell types, followed by astrocytes and oligodendrocytes.

### 2.4. Development of a Hypertonic Osmotic Shock SCI Model in a Dish

**Figure 2:**
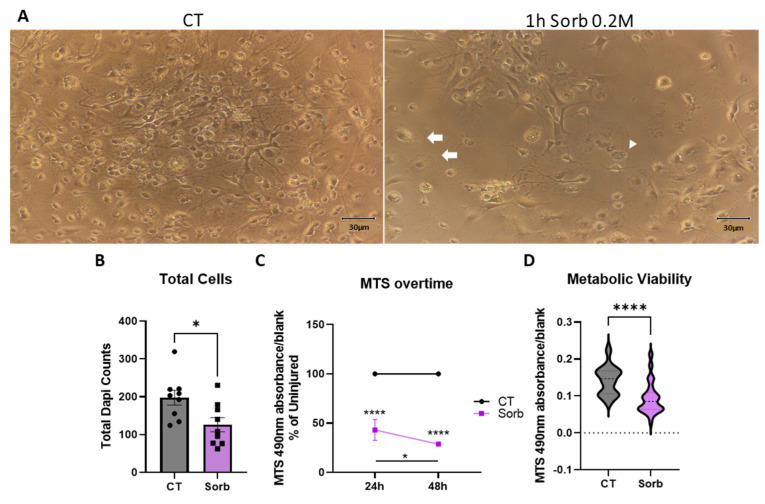
Development of a hypertonic osmotic shock SCI in vitro. (**A**) Twenty-four hours delayed bright-field microscopy inspection of cell death responses after a 1 h sorbitol insult. (**B**) Quantification of cell death responses after a 1 h sorbitol injury followed by a 48 h delay. (**C**) Progression of metabolic viability decay after a 1 h sorbitol injury at 24 and 48 h delays. (**D**) Metabolic viability after a 48 h delay of a 1 h sorbitol insult. Data are shown as mean ± SEM in (**B**,**C**) and as a violin plot with median in (**D**). Arrowhead shows apoptotic cells and full arrows show necrotic cells. Individual data points in graphs represent biological samples (cell culture wells) and are representative of three independent assays. The unpaired *t*-test was used for (**B**), mixed effects ANOVA for (**C**), and Mann–Whitney test for (**D**). * *p* < 0.05 and **** *p* < 0.0001. White arrows point to necrotic cells and white arrowhead point to an apoptotic cell.

The development of in vitro culture systems that could be applied to modeling SCI has evolved from the use of simple cultures with one cell type to complex systems involving different cell–cell interactions and three-dimensional environments in organoids or even assembloids [14,15]. While the former models are easily implemented and can be more directly scaled up through the use of high-throughput systems, they lack the complexity required to mimic the establishment of SCI-relevant pathophysiological insults. However, the latter suffer from the relative difficulty of establishment and have very high implementation costs. With that in mind, this work attempted to develop a system that would be amenable to translate complex pathophysiological insults with relative operational and cost efficiency. To achieve that, a new framework around a novel initiating pathomechanism involved in the pathophysiology of SCI was tested, with the use of a hyperosmolar sugar-alcohol to mimic the hypertonic stress seen in the early phases of the vascular injury associated with SCI [38,39]. The results of exposing the mixed spinal cord cultures to 0.2 M sorbitol for one hour are shown in Figure 2. Bright-field images taken 24 h after injury showed high amounts of cell death, as seen by the reduction in cellularity within the field-of-view and morphological signs of the presence of both apoptotic and necrotic cells [40] (Figure 2A). Confirmation of the injury impact and the association with cell death was examined by counting DAPI-stained cells and confirmed a 36% relative reduction in cell numbers 48 h after sorbitol exposure (Figure 2B). To understand the temporal dynamics of the ensuing cell death after sorbitol exposure, evaluation of the metabolic viability using the MTS test was used to follow delayed responses to injury. The data showed that although the majority of the MTS reduction happened within 24 h after injury, a continuous degenerative process still evolved until 48 h, highlighting that the model may recapitulate the progressive cell death seen in SCI in vivo (Figure 2C,D). In this context, the data showed that sorbitol-induced hypertonic stress led to sustained cell death, which rendered the mixed spinal cell culture amenable to be used as a screening platform for testing promising therapeutic strategies for SCI.

### 2.5. The Secretome from Adipose-Derived Mesenchymal Stromal Cells Is Cytoprotective Against Hyperosmolar Stress in Mixed Spinal Cord Cells

**Figure 3:**
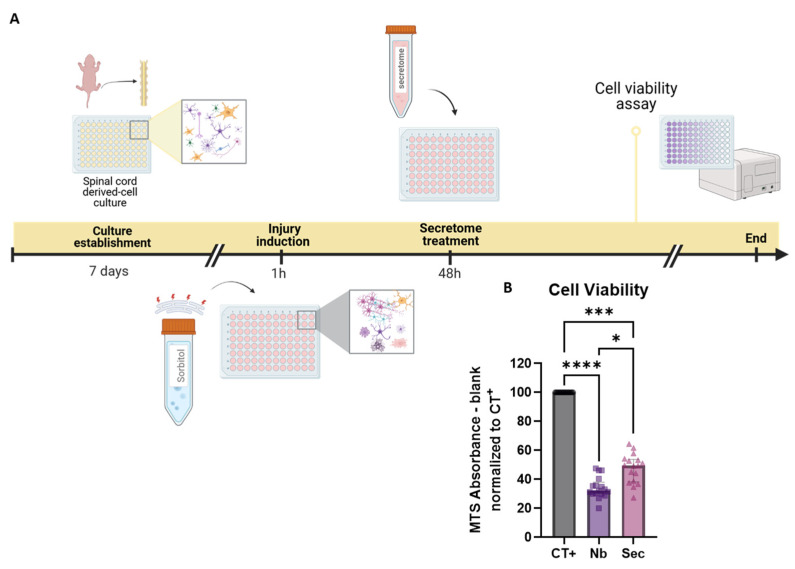
Cytoprotection assay following hypertonic osmotic shock SCI and MSC secretome treatment. (**A**) Experimental paradigm of sorbitol injury and MSC secretome treatment, followed by the MTS viability assay. (**B**) Quantification of the metabolic viability response with MTS after a 1 h sorbitol injury and 48 h secretome treatment. Data are shown as median ± IQR. Individual data points in graphs represent biological samples (cell culture wells) and are representative of three independent assays. Kruskal–Wallis test. * *p* < 0.05, *** *p* < 0.001, and **** *p* < 0.0001. Graphical objects were created with BioRender.com.

To assess the existence of a possible protective effect elicited by the secretome of adipose MSCs, the mixed spinal cord cells were subjected to sorbitol injury for 1 h, followed by 48 h of treatment with the secretome or the neurobasal medium control, as shown in Figure 3A. Analysis of the metabolic viability response after treatment revealed that the secretome-treated cells presented 28% higher MTS readings when compared to injured cells treated with the vehicle media, showing that the ASC secretome elicited significant protective effects (Figure 3B).

### 2.6. Neuronal Phenotypic Response to Sorbitol Injury and Effects of the ASC Secretome

**Figure 4:**
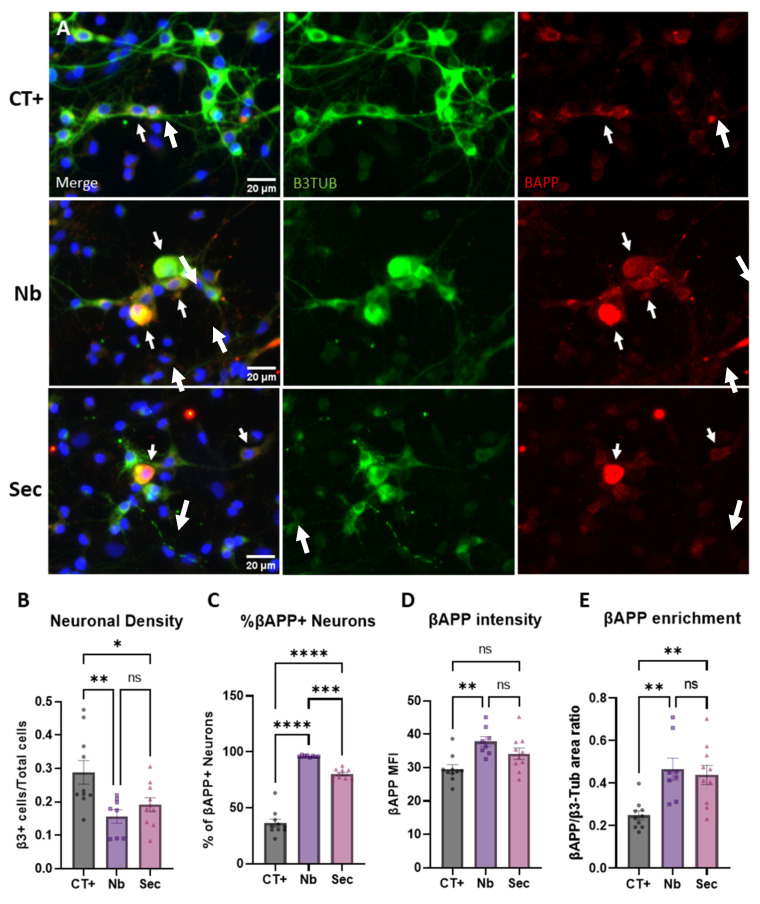
Neuronal phenotypic responses to hypertonic stress injury and effects of the hASC secretome treatment. (**A**) Representative widefield fluorescence photomicrographs of uninjured (CT+) and sorbitol injured and treated (Nb and Sec) neurons labeled with β3-tubulin and β-APP (pointed out with a white arrow). (**B**) Impact of sorbitol injury and treatments on the neuronal density of cultures. (**C**) Quantification of the percentage of neurons overexpressing β-APP. (**D**) Quantification of β-APP intensity within neurons. (**E**) Quantification of β-APP area occupancy within neurons. Data are shown as mean ± SEM. Individual data points in graphs represent biological samples (cell culture wells) and are representative of 3–4 independent assays. One-way ANOVA. * *p* < 0.05, ** *p* < 0.01, *** *p* < 0.001, **** *p* < 0.0001, and ns = non-significant.

To gain further insight into the role of the secretome from hASCs and to better understand the impact of hypertonic stress in neurons, injured cells were treated with 1x secretome immediately after the 1 h sorbitol injury, and injury-specific phenotypes were assessed by image analysis (Figure 4A). Quantification of neuronal density revealed that hypertonic injury significantly killed neurons, as seen in Figure 4B, with a near 50% reduction over the uninjured controls, and that although the ASC secretome induced a mild protective effect (−22% over the control), it was statistically non-significant. Of note, the morphologic appearance of neuronal cells in the secretome-treated group resembled more the ones of the uninjured group, with a lower frequency of large, bloated cells and a higher frequency of star-shaped cells, with axonal preservation when compared to the neurobasal medium control (Figure 4A). Fluorescence imaging analysis of the patterns of β-APP labeling demonstrated that in injured conditions, the majority of remaining neurons were positive for β-APP (Figure 4A,C and Supplementary Figure S3). The relative difference from the uninjured control showed that the upregulation of β-APP can be seen as a nuanced injury response that should be carefully analyzed.

The response seen in Supplementary Figure S3 shows the existence of a correlation between the degree of neuronal injury and the intensity of β-APP expressed in the neuronal soma. Cells with clear axonal fragmentation presented higher somatic β-APP expression (Supplementary Figure S3A–C). Additionally, according to its roles in maintaining synaptic homeostasis, β-APP expression was also found at the tips of the dendritic branches of surviving neurons (Supplementary Figure S3D). Treatment with the hASC secretome for 48 h led to a protective phenotype, maintaining a higher proportion of surviving neurons without β-APP expression (Figure 4C). Additional analysis of the relative β-APP area occupancy and intensity within surviving β3-tubulin-positive neurons highlighted similar protective responses, although not statistically significantly different from the vehicle-treated controls (Figure 4D,E).

### 2.7. Astroglial Phenotypic Response to Sorbitol Injury and Effects of the hASC Secretome

**Figure 5:**
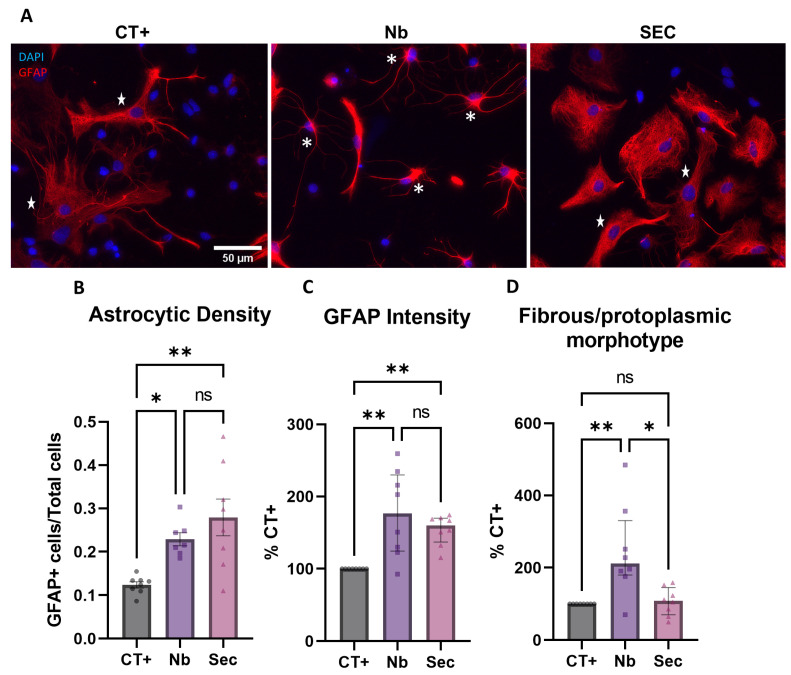
Astrocyte phenotypic responses to hyperosmolar stress injury and effects of the hASC secretome treatment. (**A**) Photomicrographs of widefield fluorescence images of GFAP+ astrocytes after injury and treatments. Fibrous morphotype is marked as (*) and protoplasmic morphotype is marked as (★). (**B**) Astrocytic density after injury and the effect of treatment. (**C**) Intensity of the GFAP signal segmented from astrocytes, normalized as a percentage of uninjured controls. (**D**) Quantification of changes in the morphometric phenotype of astrocytes. Data are shown as mean ± SEM for (**B**) and median ± IQR for (**C**,**D**). Individual data points in graphs represent biological samples (cell culture wells) and are representative of 3–4 independent assays. One-way ANOVA was used for (**B**) and the Kruskal–Wallis test for (**C**,**D**). * *p* < 0.05, ** *p* < 0.01, and ns = non-significant.

The evaluation of the astrocytic response to osmotic shock injury was performed by assessing phenotypic changes based on the density, GFAP expression, and morphology of astrocytes. It is expected that astrocytes are a major response element to osmotic perturbations, as they form neurovascular coupling structures that are relevant for controlling fluid dynamics in the brain and the spinal cord [41,42,43,44]. Our data demonstrated that after sorbitol injury, a considerable phenotypic shift was seen based on the expression patterns of GFAP, as seen in Figure 5A. A considerable rise in the density of astrocytic cells was seen after sorbitol injury, on which the secretome treatment had no effect (Figure 5B). This effect is probably derived from the massive cell death along the neuronal lineage. The analysis of GFAP intensity showed similar effects of sorbitol injury, which led to increased GFAP expression, again with no modulatory effect seen for secretome treatment (Figure 5C). Accordingly, after sorbitol injury, vehicle-treated groups presented a drastic shift in morphology, with a nearly two-fold increase in fibrous astrocytes versus protoplasmic ones, when compared to uninjured controls (Figure 5A,D). Remarkably, this morphologic phenotypic shift was completely prevented with secretome treatment (Figure 5D). Together, these results are relevant, as they confirmed that perturbations in osmotic balance are profoundly sensed by astrocytic cells, probably due to the expression of osmotic sensors, such as aquaporin proteins, and that factors contained within the ASC secretome are able to partially modulate these responses.

### 2.8. Microglial Phenotypic Response to Sorbitol Injury and Effects of the hASC Secretome

**Figure 6:**
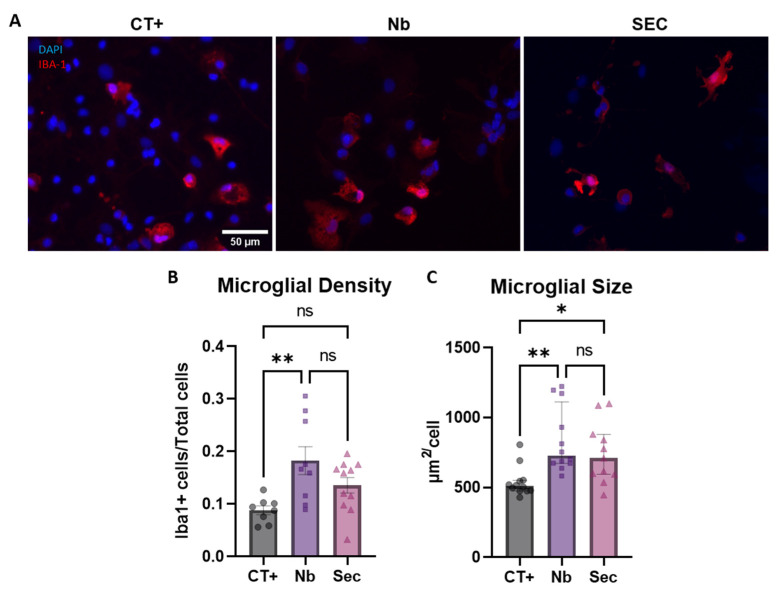
Microglial phenotypic responses to hyperosmotic stress injury and effects of the hASC secretome treatment. (**A**) Widefield fluorescence microphotographs of IBA-1+ microglia after injury. (**B**) Quantification of changes in the cellular density of microglial cells, expressed as a percentage of total cells. (**C**) Quantification of microglial cell size, expressed as the average cell size in the field of view, expressed as µm^2^. Data are shown as mean ± SEM for (**B**) and median ± IQR for (**C**). Individual data points in graphs represent biological samples (cell culture wells) and are representative of three independent assays. One-way ANOVA was used for (**B**). * *p* < 0.05, ** *p* < 0.01, and ns = non-significant.

As microglial cells are the resident immune cells of the CNS and play crucial roles in orchestrating the initial pathophysiological response after SCI, their response to sorbitol injury was also assessed (Figure 6). By staining with IBA-1 and performing unbiased, automatic quantification, it was possible to show that the sorbitol injury induced an increase in the microglial population density, as well as in its relative size (Figure 6A–C). In this context, the ASC secretome treatment for 48 h had a preventive effect in halting microglial proliferation, as its numbers were not significantly different when compared to the uninjured controls, as opposed to the vehicle-treated group (Figure 6B). In relation to morphometric adaptations, the secretome failed to elicit any protective effect when compared to either group (Figure 6C). Given the relevance of morphometric parameters and the association with microglial function, a more granular analysis of these features was performed [45].

No effects for the hyperosmotic injury in shifting either microglial solidity, perimeter, Feret’s diameter, or circularity were found (Supplementary Figure S4). This revealed that hyperosmotic injury induced only gross morphologic alterations in microglial cells. Overall, these data may show that the effects of secretome treatment on modulation of microglial reactivity to hyperosmotic stress were rather small, pointing toward other cell types as mechanistic targets.

### 2.9. Oligodendrocytes’ Phenotypic Responses to Hypertonic Stress Injury and the Effects of the hASC Secretome

**Figure 7:**
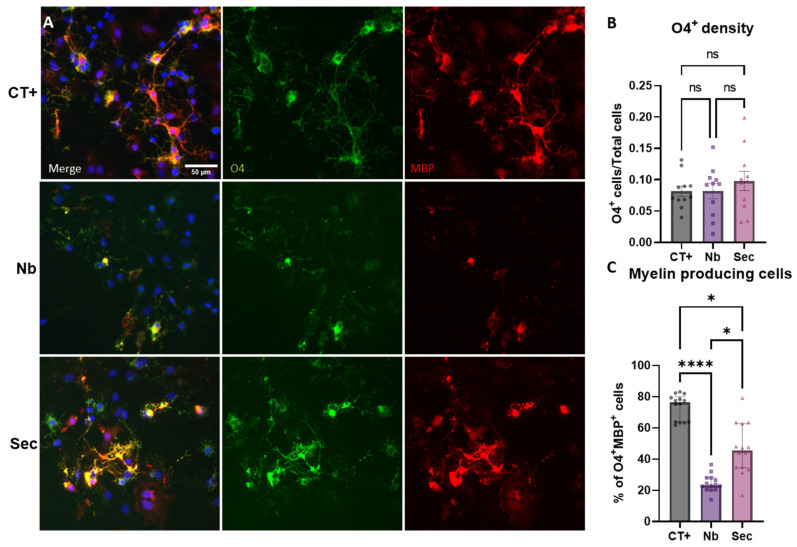
Oligodendroglial phenotypic responses to hypertonic stress injury and effects of the hASC secretome treatment. (**A**) Widefield fluorescence microphotographs of O4+ and MBP+ oligodendrocytic cells after injury and treatments. (**B**) Quantification of changes in the cellular density of oligodendroglial cells, expressed as a percentage of total cells. (**C**) Quantification of the number of O4+ cells presenting MBP+ co-labeling. Data are shown as mean ± SEM for (**B**) and median ± IQR for (**C**). Individual data points in graphs represent biological samples (cell culture wells) and are representative of three independent assays. One-way ANOVA was used for (**B**) and the Kruskal–Wallis test for (**C**). * *p* < 0.05, **** *p* < 0.0001, and ns = non-significant.

As oligodendrocytes are also key players in the pathophysiology of SCI, the response of this cell population to sorbitol injury was evaluated. Upon sorbitol injury, the quantification of cells expressing the pre-myelinating marker O4 revealed that cells maintained relatively stable numbers when normalized to the total cells in the field of view (Figure 7A,B). When the effect of sorbitol injury on the expression of MBP was assessed, it revealed that the hyperosmolar stress induced a significant reduction in the fraction of O4^+^ cells that also expressed MBP (Figure 7A,C). The cells in the injured group treated with vehicle control medium (Nb) showed a significant reduction in the area covered by O4^+^ and MBP^+^ processes, while they retained somatic expression of O4 (Figure 7A), highlighting a specific pathological response. In this specific phenotype, the ASC secretome treatment elicited a significant modulatory effect, effectively preventing the loss of MBP expression from O4^+^ cells. These data show that osmolality perturbations elicited significant responses in cells of the oligodendrocytic lineage, and that this cell population may be a cellular target of the protective effects of the ASC secretome.

### 2.10. The Secretome from hASC Modulates the Transcriptional Response of the Mixed Spinal Cord Cells to Hyperosmolar Stress

**Figure 8:**
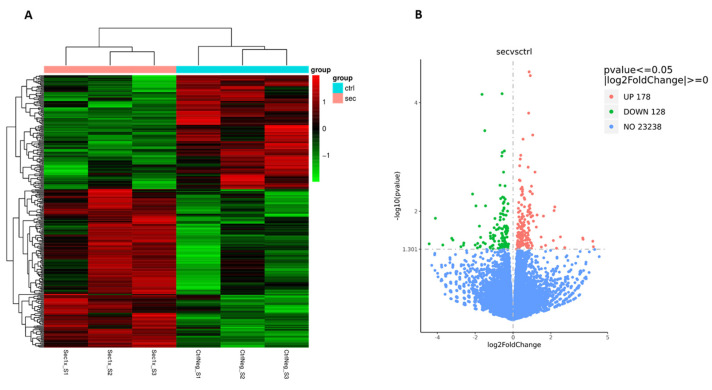
Bulk RNA sequencing of the SCI culture after a 24 h hASC secretome treatment. (**A**) Hierarchical clustering of the secretome-treated (Sec) and vehicle control (CtrlNeg) groups, based on their differentially expressed genes. Color code represents log2(FPKM + 1) expression values. (**B**) Volcano plot of gene expression dispersion considering a log2FoldChange and statistical significance of −log10 (*p*-value).

To acquire a mechanistic insight into the sorbitol injured spinal-cord-derived culture response to secretome treatment, a transcriptional analysis was employed (Figure 8). mRNA sequencing was performed on both treatment (Sec) and vehicle control (CtrlNeg) groups after a 24 h secretome treatment to capture early gene expression changes. The hierarchical clustering of the differentially expressed genes from each sample revealed the existence of several gene cluster sets, reflecting a significant gene expression shift upon secretome treatment (Figure 8A). The secretome transcriptional impact could also be confirmed in a screening for the significantly different expressed genes from both groups, revealing a total of 347 genes being differently expressed (FDR < 0.05). Of these, 178 genes were being upregulated and 128 downregulated after secretome treatment (Figure 8B).

**Figure 9:**
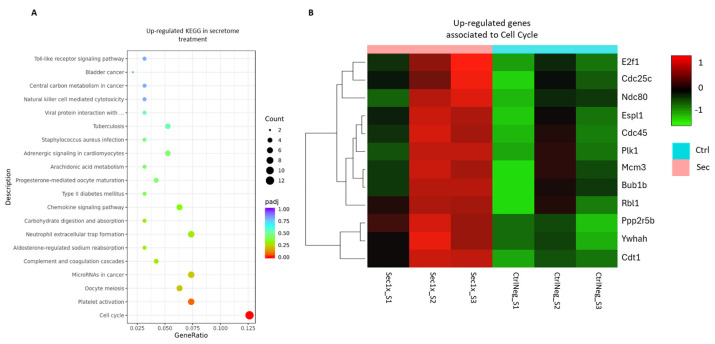
Enrichment analysis of the differently upregulated genes in the SCI culture following the hASC secretome treatment. (**A**) Pathway enrichment analysis of the upregulated genes upon hASC secretome treatment, correlating them with Kyoto Encyclopedia of Genes and Genomes (KEGG). (**B**) Hierarchical clustering of secretome-treated (Sec) and vehicle control (CtrlNeg) groups, based on their significantly upregulated genes related to cell cycle pathways. Color code represents log2(FPKM + 1) expression values.

To associate the identified differentially expressed genes with variances in biological functions and pathways, an enrichment analysis was carried out. Kyoto Encyclopedia of Genes and Genomes (KEGG) pathway enrichment analysis revealed a significant over-representation of cell cycle genes related to hASC secretome modulatory functions (Figure 9A,B).

**Figure 10:**
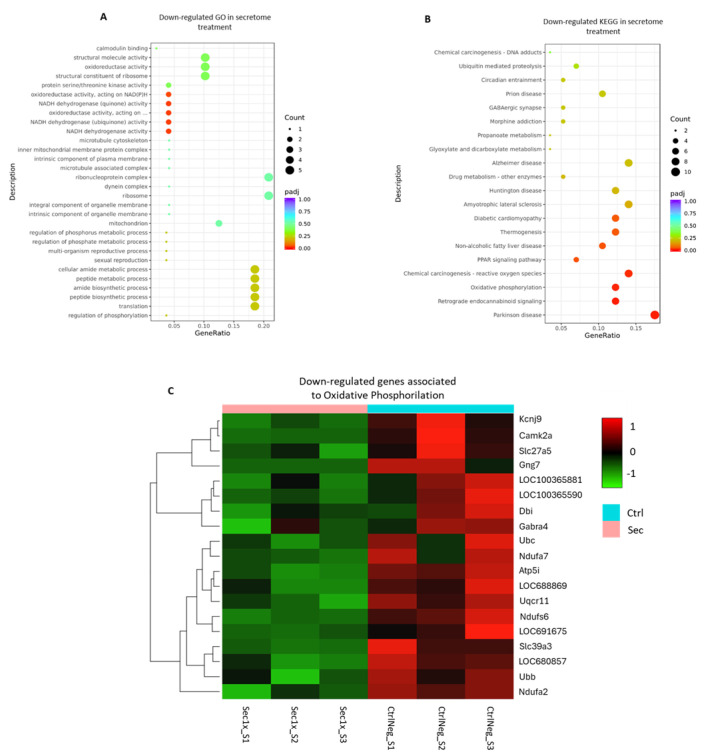
Enrichment analysis of the differently downregulated genes in the SCI culture following the hASC secretome treatment. (**A**) Gene ontology analysis of the downregulated genes upon hASC secretome treatment, classifying them into three main branches: biological processes, molecular functions, and cellular components. (**B**) Pathway enrichment analysis of the downregulated genes upon hASC secretome treatment, correlating them with the Kyoto Encyclopedia of Genes and Genomes (KEGG). Significance was considered in *p*-value adjusted for multiple comparisons < 0.05. (**C**) Hierarchical clustering of the secretome-treated (Sec) and vehicle control (CtrlNeg) groups, based on their significantly downregulated genes related to the oxidative phosphorylation pathway. Color code represents log2(FPKM + 1) expression values.

This treatment also revealed a downregulation of pathways associated with oxidative phosphorylation and reactive oxygen species (ROS) production (Figure 10A) in gene ontology (GO) analysis. Correspondingly, significantly downregulated pathways found within KEGG analysis were also linked to mitochondrial function and phosphorylation processes (Parkinson’s disease pathways), oxidoreductase activities, ubiquinone metabolism, and ion channel regulation (PPAR and retrograde endocannabinoid signaling; Figure 10B). The magnitude of effect of these differentially expressed pathways is further supported in Figure 9B and Figure 10C, where the hierarchical clustering of the enriched-pathway-associated genes is illustrated, based on their expression patterns across sample groups.

## 3. Discussion

Innovations in cell culture models of SCI will be instrumental for the success of translational platforms aiming to find effective new therapies [17]. In this work, an innovative in vitro model of SCI pathophysiology was developed using a primary mixed spinal cord cell culture isolated from rat pups. The cellular model was composed of the major cell types encountered in the spinal cord parenchyma and presented unique advantages in comparison to other models regarding feasibility, reproducibility, and cost-effectiveness [46]. The model can be implemented in any biomedical laboratory equipped with basic cell culture equipment, as it does not require specific hardware. The cell culture system was characterized based on expression of lineage-specific immunocytochemical markers, which confirmed the presence of neurons, astrocytes, oligodendrocytes, and microglial cells. Importantly, as the model was established in only seven days, a relatively early phenotype in terms of cell differentiation was present, enabling its use for screening of differentiation-inducing compounds [47]. At seven days post-establishment, the model was demonstrated to recapitulate after treatment with a hyperosmolar sugar-alcohol able to elicit hyperosmotic stress, key hallmarks of pathophysiological responses seen in complex SCI environments [21]. The sorbitol injury induced marked responses in the cell culture, as seen by a 65% reduction in the metabolic viability of cells after 1 h of contact and a 48 h delay. Importantly, the cell loss reflected in the MTS readings was shown to be gradual, demonstrating that the model recapitulated a graded wave of cell death. Such feature is important for the screening of therapeutic agents that target neuroprotective or neuroregenerative mechanisms, as they depend on the persistence of viable cells as mechanistic targets [48]. This is the first study to report the application of sorbitol as an inductor of hyperosmotic stress in spinal cord cultures, with the intent of inducing a “SCI in a dish” model. The analyses of cell phenotypes along specific lineages after injury showed that the established model produced marked phenotypic shifts in neuronal, astroglial, microglial, and oligodendrocytic populations. Specifically, over 45% of neurons died in the first 48 h of injury, with the vast majority of the remaining ones expressing the axonal injury and stress marker β-APP [49]. The occurrence of such phenotypes, as well as the presence of axonal beading and fragmentation (although not quantified), highlighted that the model could also be employed in studies assessing axonal transport and proteostatic mechanisms of degeneration [21,50].

In the astrocytic population, a major phenotypic shift was also observed. The morphology of astrocytes in the presence of hyperosmotic stress largely resembled that of fibrous astrocytes, presenting high levels of GFAP expression. The relative difference from the control in the abundance of fibrous astrocytes was more than two-fold. Interestingly, other studies assessing hyperosmolar stress responses in purified human astrocytic cultures have revealed a relatively smaller pathological phenotype after a much longer sorbitol exposure [21]. These differences are likely explained by the use of a complex cell culture containing other cell types that together elicit a reactive phenotype in astrocytes [51].

In the microglial population, a significant increase in cell density alongside increments in cell size were found, indicative of a classic reactive phenotype upon cell stress and sterile inflammation [52,53], whereas in the oligodendrocytic population, although there were no changes in the amount of O4^+^ cells after injury, the amount of cells expressing MBP was significantly reduced. This can also be seen as an injury-specific response, as reduced myelination has been shown to be one of the initial hallmarks following SCI [54]. Of note, a deeper analysis of microglial morphometric parameters failed to reveal an injury-specific phenotype. This demonstrated that the effects of hyperosmotic injury were translated only by major shifts in morphology, which were missed in analyses of more granular morphologic traits.

Taking all of this into account, in vitro models of SCI are often developed as tools to simplify the complexity of its pathophysiologic mechanisms, enabling the interrogation of specific questions with less confounders [55]. If these models reproducibly recapitulate pathologic mechanisms of SCI, they can be valuable for the study of new therapeutic compounds [17]. In this context, there are four major types of in vitro systems for SCI research, which include several types of two-dimensional systems of cell lines or primary cultures, three-dimensional organotypic cultures, specific microfluidic devices, as well as organoids and assembloids [15,56,57,58,59,60,61]. Each of these systems have been employed differently in the investigation of pathophysiologic events relevant to SCI. For instance, monolayers of cells have been used mostly in models of neurotoxicity induced by reactive oxygen species [62,63], pro-inflammatory environments [51,64], and growth factor deprivation [65,66], but have also been employed to interrogate neuronal growth potential in response to SCI-specific inhibitory environments [67,68]. While organotypic cultures have been used for the study of axonal growth [29,69,70] or multi-cellular responses to mechanical trauma or excitotoxicity [59,71,72], microfluidic chambers have been successfully employed for the study of axonal growth and regeneration mechanisms [60,73,74,75].

Although these models have shown promise in addressing these specific questions, apart from thick organotypic cultures, they often lack the cellular complexity and 3D cytoarchitecture of the spinal cord, reducing the translatability of the information generated. Lately, cell culture systems using organoids have been successfully generated to recapitulate key hallmarks of spinal cord development and function [46,61]. Fusion of different organoids was also performed to generate a cortico-motor assembloid that is able to elicit motor responses upon electrical stimulation [15]. If future applications of these multi-dimensional models are directed to the investigation of traumatic injury responses, large amounts of relevant data may be generated, holding great potential for SCI research. However, several aspects are important to be discussed if these advanced models are to be embraced on a large scale. The first issue has to do with the relative cost of implementation, which will hinder broad adoption by not-so-well-funded laboratories [76]. Also, the complexity in developing and maintaining these systems adds variability, as they often require specific hardware and trained personnel, which further limits their applications [77].

In this context, by presenting both glial and neuronal populations, the model proposed in this study circumvents the reductionist problems found with simple 2D primary cultures or cell lines. This added cellular complexity represents a leap forward in terms of enhanced translatability given the increased quantity of questions that can be interrogated, as well the quality of multi-dimensional data that can be generated. These features, coupled with the feasibility, reproducibility, and cost-efficiency, places the model presented in this study in between simple 2D cultures and complex 3D systems [18].

Regarding the choice of injury induction method, although traditionally not studied in SCI in vitro models, the relevance of vascular mechanisms is well known from in vivo studies and is taught to be a key initiating factor of the secondary mechanisms of injury [4,5,78]. Hence, modeling osmotic perturbations may provide a link between the start of the injury mechanisms and the multi-cellular phenotypic responses induced by vascular disruption in spinal cord cells. As it has been demonstrated, the most important osmolyte in the blood is albumin, which helps maintain osmolality around 290 mosmol/kg [79]. Upon SCI, extravasation of blood components, such as albumin, fibronectin, and Ca2+, into the spinal cord is a well-known phenomenon that correlates with blood–spinal cord breakage and injury severity, which peaks early after injury [80,81,82,83]. Although investigations measuring increases in local osmolality in the spinal cord parenchyma after SCI have not yet been conducted, it can be expected that this increase in the concentration of these blood-born compounds raises local osmolality, which may act as an injury mechanism elicited by vascular injury [84]. Accordingly, our study provided evidence to support the notion that increases in osmolality indeed lead to injury manifestations in neuronal and glial cells isolated from the spinal cord of mammals. In fact, the osmolyte sorbitol has been used as a stressor in neuronal and glial cell cultures, including motor neurons differentiated from induced pluripotent stem cells, leading to oxidative stress and disrupted RNA processing, two mechanisms associated with spinal cord injury [21,25,26]. Although we believe that inducing hyperosmolar perturbation in spinal cord cells is a viable way to model SCI mechanisms in vitro, several limitations exist.

For instance, it is worth mentioning that the inflammatory environment characteristic of the secondary injury cascade events will never be fully recapitulated by the use of sorbitol in this in vitro SCI model. This is a limitation originating from the lack of the immune system response and, specifically, the absence of infiltrating immune cells (monocytes and lymphocytes), as well as the lack of complexity of in vivo three-dimensional cytoarchitecture that mediates these interactions [85]. In addition, our microscopical evaluations highlighted that microglial cells, and astrocytes to a lesser degree, presented different morphologies to what is seen in vivo. We believe this can be explained by the differences in substrate stiffness and elasticity in poly-d-lysine-coated substrates [86,87,88,89]. Nevertheless, their morphological parameters were altered following hyperosmotic stress, demonstrating the robustness of the model.

In line with this, the injury model was employed to verify if the mesenchymal stromal cell secretome was able to modulate these injury-induced phenotypic manifestations. Remarkably, protective effects were seen for the ASC secretome when assessing the metabolic viability of the cells after injury. The assessment of the secretome performance on the modulation of lineage-specific injury response phenotypes was also assessed.

The secretome was effective in limiting β-APP expression in the surviving neurons, which can directly reflect a stress-protective effect. This result correlates with what was previously known about the neuroprotective properties of the ASC secretome in counteracting axonal morphological changes, such as beading, in models of oxidative stress [90]. This increase in β-APP accumulation seen in our model may be derived from disruption of axonal transport induced by macromolecular crowding, which is a feature of hyperosmotic stress [21]. In this context, the mechanisms behind the secretome effect are likely derived from the presence of neuroprotective and antioxidant factors, such as BDNF, GDNF, DJ1, thioredoxin, and peroxiredoxin 1 [91].

The secretome effect was also evidenced in the significant attenuation of the astrocytic morphological shift witnessed after hyperosmotic stress. It is known that hyperosmolar conditions lead to a broad remodeling of the cytoskeletal machinery of actin filaments, starting as early as 15 min after hyperosmolar stress [92]. The fact that the ASC secretome prevented this phenotypic change observed at 48 h after insult shows that the secretome played a role in the activation of osmo-adaptation processes. Accordingly, the hASC secretome enhanced the expression of a key osmo-adaptation gene product (fxyd2) by a 1.16-fold change (*p* = 0.0019), which has been highly implicated in adaptation to osmotic imbalances in several physiological contexts [93,94,95]. This protective effect may be associated with a possible reduction in the reactive astrogliosis process and establishment of a neurodegenerative environment in SCI [96]. Although microglial cells responded to the hyperosmolar injury with an apparent increase in the proliferative and morphometric response, the ASC secretome did not induce a protective effect, indicating that the factors contained within the secretome may be specifically modulating other cellular targets. Lastly, the myelination-associated markers within the injury treatment paradigm revealed that the hASC secretome had the potential to modulate the injury effect by enhancing the number of pre-myelinating oligodendrocytes expressing MBP. This beneficial effect could be attributed to several hASC-conditioned medium components (such as IGF-1, BDNF, or TGF-β) that support remyelination and differentiation of OPCs into myelinating OLs [29,97,98,99,100].

As the transcriptional landscape induced by hyperosmolar injury has been shown to reveal possible mechanistic targets for new treatments, an RNA-sequencing experiment was conducted. The functional enrichment analysis of these genes revealed a significant upregulation of cell cycle pathways upon secretome treatment.

Twelve genes with functions in cell proliferation and division were found to be upregulated, highlighting Mcm3, essential for DNA replication [101], and Cdc45, which has a role in its initiation [102]. Also, several other key genes were found for regulatory processes and checkpoints in cell division, such as Espl1, essential for a proper chromosome segregation [103], or Plk1, related to ATP binding and mitotic progression [104], which is upregulated in response to an increase in proliferation. Notably, another cell cycle regulating gene, E2f1, has been discovered to have a particular role in cell survival and is involved in modulation of metabolism by modulating metabolic shifts from oxidative phosphorylation to glycolysis [105]. In the context of our data, the modulation of these gene sets indicated that the secretome acted through the modulation of osmo-adaptation processes, which culminated in the preservation of cell cycle responses to injury. Given that the arrest in cell cycle progression is the final cellular process elicited by osmotic stress, as shown by several reports [27,106,107], this secretome-mediated response is relevant also in the context of SCI in vivo. The maintenance of cellular viability is crucial to preserve cells that are the target of neuroprotective and regenerative therapeutic applications [8,108,109].

Overall, the upregulation of the mentioned cell cycle functions suggested that the secretome maintained a transcriptional landscape conducive to cell survival and metabolic viability, supporting the MTS assay findings. Functional enrichment analysis of the downregulated GO terms and KEGG pathways revealed a significant downregulation of several correlated pathways and molecular functions associated with metabolic adaptations and responses to stress.

For instance, a significant decrease in NADH dehydrogenase and oxidoreductase activity-related genes, such as Ndufb7, played an important role in the downregulated oxidative phosphorylation pathways [110]. The downregulation of genes involved in this pathway indicated a clear metabolic shift toward glycolysis upon secretome treatment by protectively reducing mitochondrial activity to consequently decrease the production of ROS, which may be upregulated in the sorbitol injury context. Interestingly, this analysis also revealed a downregulation of chemical carcinogenesis genes related to ROS production in the treatment group, such as Uqcr11, which have a role in the mitochondrial electron transport chain and, consequently, oxidative phosphorylation [111]. Downregulated genes related to Parkinson’s disease and retrograde endocannabinoid signaling, such as Camk2a, also have a role in phosphorylation, oxidoreductase, and ion channel activities [112]. All of these contribute to mitochondrial respiration energy production, corroborating the previous results and mediating the possible antioxidant properties of the secretome, ultimately leading to cellular protection [69]. Lastly, this analysis also showed a downregulation of the peroxisome proliferator-activated receptor (PPAR) pathway after treatment. This could be translated into several cellular impacts, such as reinforcing the existence of a metabolic shift to reduce ROS production, since this pathway regulates the fatty acid oxidation in lipid metabolism [113]. On the other hand, this could also mean an induction of a cellular strategy to avoid lipid peroxidation, resulting from the interaction of the ROS byproduct of the lipid metabolism with the unsaturated fatty acids in cell membranes [114]. In this manner, it is expected that cellular stability and integrity are enhanced as a response to the secretome under the osmotic stress condition.

Gathering all these results together, the hASC secretome could be acting in response to both osmotic and resultant oxidative stress by inducing the maintenance of intracellular redox homeostasis, leading to a metabolic reprogramming and antioxidant effect. At the same time, it seems to sustain cell cycle progression by enhancing the expression of cell-cycle-related genes.

To conclude, this work proposed the development of a novel SCI in vitro model for the assessment of multi-cellular interactions in a hyperosmolar injury, in a reproducible and effective manner. The use of the physiological stressor sorbitol successfully induced relevant phenotypical responses that correlated with the pathophysiology of SCI. This model was applied to assess the role of the hASC secretome as a cytoprotective agent and its modulatory function on the phenotypical manifestations of single-cell populations. It was found that the hASC secretome induced significant preservation of cellular viability after injury, which was associated with positive modulatory functions in the neuronal stress response, as well as glial cell reactivity. Mechanistically, transcriptomic analysis revealed that the secretome modulated genetic programs associated with reduced oxidative damage and the preservation of cell cycle processes, which are linked to major pathophysiological hallmarks of hyperosmotic stress.

## 4. Materials and Methods

### 4.1. Animals

P5 newborn Wistar rats of both sexes (Charles River; RRID_RGD_737929) were used for in vitro experiments. The animals used in this study were maintained under standard laboratory conditions (12 h light/12 h dark cycle, 22 °C, relative humidity of 55%, ad libitum access to standard food and water). Rats were maintained 2 per cage. All experiments were approved by the Portuguese Regulatory Entity (DGAV 022405) and conducted in accordance with the local regulations on animal care and experimentation (European Union Directive 2010/63/EU).

### 4.2. Spinal Cord Cells’ Isolation and Maintenance

Spinal cord cells were isolated from P5 newborn Wistar rats (RRID_RGD_737929), as described previously, with adaptations [115]. Briefly, the rats were euthanized by decapitation, and the spinal columns were harvested with sharp scissors along the thoraco-lumbar region. The spinal cord tissue was isolated using hydraulic extrusion, as previously demonstrated [32]. The spinal cords were enzymatically digested in a dissociation solution (0.25% trypsin, Gibco, Billings, MT, USA, Cat. No. 25300062) for 3 rounds of 5 min at 37 °C, followed by mechanical dissociation. Enzymatic digestion was stopped by adding inactivation media DMEM-F12 (Sigma, St. Louis, MO, USA, Cat. No. D5648) with 10% FBS (S0615, Sigma-Aldrich, St. Louis, MO, USA) to the cell suspension. The cell suspension was passed through a 70 µm strainer and was centrifuged at 175 g for 10 min without brakes to form a cell pellet. The pellet was resuspended in culture medium, and a 1:1 dilution of Trypan Blue (Sigma, St. Louis, MO, USA, Cat. No. T8154) was prepared for cell counting under a light microscope, the Olympus BX51WI Fixed Stage Upright Microscope (RRID:SCR_023069), using a Neubauer chamber (Marienfeld, Harsewinkel, Germany, Cat. No. 0640010). A density of 31 × 10^3^ cells/cm^2^ was used for each well of a 96-well plate. Cells were plated in poly-D-lysine-coated wells using plating medium composed of DMEM-F12, with 1% L-GLUT, 1% PenStrep, and 10 nM GDNF. After the initial 24 h, the medium was changed for the maintenance medium every 48 h, composed of neurobasal with 1%L-GLUT, 1% PenStrep, 2% B27, and 10 nM GDNF. The cells were maintained in culture medium at 37 °C with 5% CO_2_ for 7 days in vitro (DIV) for characterization and for 9 days when injured with sorbitol and treated with hASC secretome. Details of the experimental paradigms for cell cultures can be seen in Supplementary Figure S1.

### 4.3. hASC Cell Culture

Human adipose tissue-derived mesenchymal stromal cells (hASCs, lot:080313), kindly provided by Professor Jeffrey Gimble (Obatala Sciences LLC, New Orleans, LA, USA), were cultured according to previously established protocols in α-MEM medium (Invitrogen, Waltham, MA, USA) supplemented with 10% FBS (S0615, Sigma-Aldrich, St. Louis, MO, USA) and a 1% antibiotic–antimycotic mixture (Invitrogen) [116].

### 4.4. Cell Density Quantification

Cells had their media removed and were fixed with 4% paraformaldehyde (PFA; PanReac, Castellar del Vallès, Barcelona, Spain) for 15 min at room temperature (RT°), followed by staining with DAPI at 0.1 μg/mL (Invitrogen) for 30 min. After 2 washes in 1× phosphate-buffered saline (PBS), cells were imaged in 100 µL of PBS using a using a motorized widefield IX81 inverted microscope (Olympus, Shinjuku, Tokyo, Japan) equipped with a 2× objective. Images were analyzed in the Fiji software 2.9.0 [117] by nuclei segmentation using Otsu thresholding [118] followed by watershed processing. Quantification was performed with the Analyze Particles plugin using a minimal size filter of 30 µm.

### 4.5. Assessment of Metabolic Viability

Following the first or the second day of injury with sorbitol, media were removed, and cells were incubated in normal culture conditions for 3 h with a 1:5 dilution of MTS reagent (CellTiter 96^®^, Promega, Madison, WI, USA) in DMEM (Sigma) without FBS. Afterwards, the media were collected and read in three technical replicates per well using a microplate reader (Infinite^®^ 200PRO, Tecan, Männedorf, Zurich, Switzerland) at 490 nm.

### 4.6. Immunofluorescence and Lineage-Dependent Characterization

For immunofluorescence staining, cells plated on poly-D-lysine-coated 13 mm glass coverslips were fixed, as previously described, and permeabilized and blocked for 60 min in 1× PBS with Triton X-100 (0.3% PBS-T) and 10% newborn calf serum (NBCS, ThermoFisher Scientific, Waltham, MT, USA). Then, primary antibodies were added in the same medium but with 4% NBCS at RT° for 4 h: mouse anti-β3-TUB, rabbit anti-β-APP, mouse anti-MAP2, rabbit anti-GFAP, rabbit anti-IBA1, rat anti-MBP, mouse anti-O4, and rabbit anti-DCX, according to adequate combinations, as shown in Supplementary Table S1. Subsequently, the samples were washed with PBS and incubated at RT° with the secondary antibodies for 2 h in the same incubation medium: Alexa Fluor 488 goat anti-mouse, Alexa Fluor 488 goat anti-rabbit, or Alexa Fluor 647 goat anti-rat (1:1000, Invitrogen) were used. Cells were washed with PBS and, finally, a counterstaining with DAPI (1 μg/mL, Invitrogen) was performed for 10 min at RT°. SuperFrost slides were mounted using Permafluor (Thermo Fisher Scientific, Waltham, MT, USA) and images were acquired at 20× magnification using the IX81 fluorescence microscope (Olympus). For analysis, unique segmentation and quantification strategies were employed to unbiasedly estimate cell proportions within our cultures using Fiji [117]. For neuronal cell density, β3-tubulin was used, as it is an adequate pan-neuronal marker labeling early-born neurons to mature neurons. Neuronal segmentation was performed after a subtract background processing, using the rolling ball method with a 30-radius threshold [119] and an automatic default thresholding. Grouped neurons were separated by watershed, and the counts were acquired with the Analyze Particles plugin with a minimal 15 µn size filter. The same strategy was used for microglial cells based on the IBA-1 marker. For astrocytes, cell nuclei were segmented first, using an automatic global Huang threshold [120], followed by a watershed for separation of doublets. The nuclei mask was then superimposed onto the GFAP image using the transparent-white function, which enables visualization of the GFAP signal inside transparent DAPI masks. Large groups of GFAP+ pixels were segmented with an auto-global default threshold and counted with the Analyze Particles plugin, with a minimal size filter of 17 µm and minimum of 0.15 circularity counted as astrocytes. A similar strategy was used to quantify oligodendroglial cells based on O4 expression; however, the settings for Analyze Particles were size filter 15–1500 µm with 0.45–1.00 circularity. All quantifications were normalized by the total number of DAPI cells in the FOV.

### 4.7. Sorbitol Hyperosmotic Injury

As changes in extracellular osmolality have been linked with the initiation of several pathological processes, including inflammation [121] and neuronal stress [21], we used sorbitol as an osmolyte to induce hyperosmolar stress. Osmotic stress was induced using sorbitol at a 0.2 M concentration diluted in maintenance media without growth factors for 1 h, under standard culture conditions. This condition roughly doubled the osmolarity of neurobasal media, which was 260 mOsm. Cells had their media replaced, returning to physiological osmolarity levels, and outcome measures were assessed as previously described after either 24 or 48 h.

### 4.8. Secretome Collection and Treatments After Injury

Cell maintenance and secretome collection were carried out according to previous work with slight modifications [116]. Briefly, ASCs at passage 6 (P6) were maintained in fresh medium in the mentioned conditions for 72 h. Thereafter, the medium was removed for the cells to be washed 5 times with phosphate-buffered saline (PBS) without Ca^2+^ and Mg^2+^ ions (Invitrogen, USA), preventing protein contamination of the conditioned ASC medium. Following this step, cells were conditioned for 24 h in the same incubation conditions in neurobasal medium (Nb Thermo Fisher Scientific, Waltham, MT, USA) with 1% P/S, adapted to the in vitro applicability. In this period, where factors and vesicles were secreted, the resulting conditioned medium/secretome was collected and centrifuged at 1000× *g* for 10 min to remove cell debris. After supernatant collection and a snap-freeze in liquid nitrogen, the secretome was stored at −80 °C to be preserved for posterior use.

After the 1 h sorbitol treatment, the injury medium was aspirated, and the secretome from hASCs and the NB control was added for a 48 h treatment.

### 4.9. Image Analysis of Injury-Induced Phenotypic Changes

For characterization of the phenotypic responses to injury and effect of treatments within our cultures, cells were seeded on poly-D-lysine-coated 96-well plates and imaged, as mentioned previously. The analysis of each combination of phenotypical markers started with a batch processing analysis of cellular densities based on cell nuclei stained for DAPI. Briefly, 8-bit images were subjected to fixed, global-intensity-based thresholding (Fiji’s default algorithm) for segmentation. Separation of connected nuclei was performed with a watershed algorithm, with nuclei numbers and morphometric characteristics being retrieved via a particle analysis algorithm using size exclusion and circularity parameters for filtering. For the quantitative analysis of the neuronal responses to injury, quantifications of both total neurons and the profile of β-APP (beta-amyloid precursor protein) expression were assessed. Initially, for normalization purposes, the total cell numbers within each image were retrieved with the DAPI processing macro mentioned above. Next, the total number of neurons was assessed based on the pan-neuronal marker (β3-tub) expression. Neuronal areas were retrieved after segmentation for normalization purposes. Cells were counted after segmentation with a local thresholding algorithm (adaptive thresholding). Adaptive thresholding has increased performance vs global thresholding when images have inhomogeneous pixel intensity distributions [122]. Quantification was performed using the cell-counter plugin. To confirm expression patterns, toggling between channels was performed with the channels tool. Only nuclei-containing β3-tub+ cells were counted. Next, the same information was retrieved from the β-APP channel, and β-APP+ neurons were counted on a composite image using the cell-counter plugin. For analysis of expression intensity, another macro was employed. Measurements of area occupancy and intensity of β-APP were retrieved from segmented masks of the β3-tub signal. Such analysis allowed for the quantification of the enrichment of β-APP within each single neuron, allowing for a more thorough analysis of the injury phenotype. For the glia cell response to injury, firstly, we started by retrieving the densities of microglial cells (IBA1+). The cell cytosol was segmented using automatic global thresholding (Otsu algorithm), where only the masked microglial areas were superimposed onto the DAPI channel. Then, only the DAPI nuclei contained inside microglial cytosols were quantified and expressed as a percentage of total DAPI nuclei. For astrocyte phenotypic shifts’ quantification, manual counting was performed using the cell-counter plugin using two classes based on the morphologic appearance of fibrous (fine, less numerous, and strong processes) or protoplasmic astrocytes (ultra-fine, more numerous, and less strong, with a bushy appearance). Oligodendrocytes were also counted with the cell-counter plugin, using 2 classes to represent O4+ only and O4+ and MBP+ cells.

### 4.10. RNA-Sequencing Analysis After Sorbitol Injury

After 24 h hyperosmolar injury induction, total RNA was extracted for bulk mRNA sequencing. Briefly, mRNA was isolated using the miRNeasy Micro Kit (Qiagen). RNA quality and integrity was assessed both by Nanodrop and Bioanalyzer, and only samples with 260/280 and 260/230 absorbance ratios 1.8–2 and RIN > 8.5 were sequenced. RNA sequencing was performed by (Novogene Company, Ltd., Beijing, China). as a service, including bioinformatic analysis. The sequence library (mRNA with polyA enrichment) was prepared using the NEBNext^®^ UltraTM RNA Library Prep Kit for Illumina^®^ (New England Biolabs) and sequenced on an Illumina NovaSeq using a paired-end 150 bp system (6 Gb per sample). Raw data were subjected to quality control to remove adapters’ contamination, reads containing N > 10%, and reads with a score of over 50% of bases below five. Alignment to the reference genome was performed with HISAT2. The distribution of gene expression levels, FPKM, and heatmaps with correlation coefficients was compared. Differential expression analysis was performed using the DESeq2 package in R. To unravel biological functions or pathways significantly associated with differentially expressed genes (DEGs), functional enrichments were calculated using clusterProfiler software (GO enrichment and KEGG). The *p*-values in dot plots were given after Benjamini–Hochberg correction for multiple comparisons.

### 4.11. Statistical Analysis

Statistical analysis was performed using GraphPad Prism (version 9). All datasets were tested for normality distribution with the Shapiro–Wilk test and by the analysis of distribution histograms, as well as the values of skewness and kurtosis, prior to any statistical analysis. Data were expressed as mean ± standard error of the mean (SEM) unless otherwise stated in the figure legends. Homogeneity of variances were measured using the Brown–Forsythe test. A one-way ANOVA was used to assess the differences between three groups and the Student’s *t*-test was used for comparison of means between two independent groups. Equivalent non-parametric tests were employed, namely, the Kruskal–Wallis or the Mann–Whitney tests. For all analyses, upon confirmation of significant main effects, differences among group means were analyzed using the Tukey test for ANOVA or Dunn’s test for Kruskal–Wallis. A value of *p* < 0.05 was considered statistically significant.

## Data Availability

The data presented in this study are available upon request from the corresponding author.

## Supplementary Materials

The following supporting information can be downloaded at: https://www.mdpi.com/article/10.3390/ijms26073298/s1.
