# Supplementary material for: Modeling Spinal Cord Injury in a Dish with Hyperosmotic Stress: Population-Specific Effects and the Modulatory Role of Mesenchymal Stromal Cell Secretome
Source: Int J Mol Sci. 2025 Apr 2;26(7):3298. doi: 10.3390/ijms26073298 (PMC11989751; doi:10.3390/ijms26073298)
Supplement: Supplementary file 1 [file ijms-26-03298-s001.zip › ijms-3458113-supplementary.pdf]

IJMS – Manuscript

Modelling Spinal Cord Injury in a Dish with Hyperosmotic Stress: Population specific effects and the modulatory role of Mesenchymal Stromal Cell Secretome

Supplementary Files

Jonas Campos [1,2#], Ana Teresa Sousa [1,2#], Luís S. Fernandes [1,2], Jorge R. Cibrão[1,2], Tiffany S. Pinho[1,2], Sofia C. Serra [1,2], Nuno A Silva [1,2], Adina T Michael-Titus [3] and António J Salgado [1,2*]

1 Life and Health Sciences Research Institute (ICVS), School of Medicine, Campus de Gualtar, University of Minho, 4710-057 Braga, Portugal

2 ICVS/3B's—PT Government Associate Laboratory, 4805-017 Guimarães, Portugal

3 Centre for Neuroscience, Surgery and Trauma, The Blizard Institute, Barts and The London School of Medi-cine and Dentistry, Queen Mary University of London, London E1 2AT, UK

# These authors contributed equally to this work.

* Correspondence: asalgado@med.uminho.pt; Tel.: +351-253-60-49-47

Supplementary Figure 1.

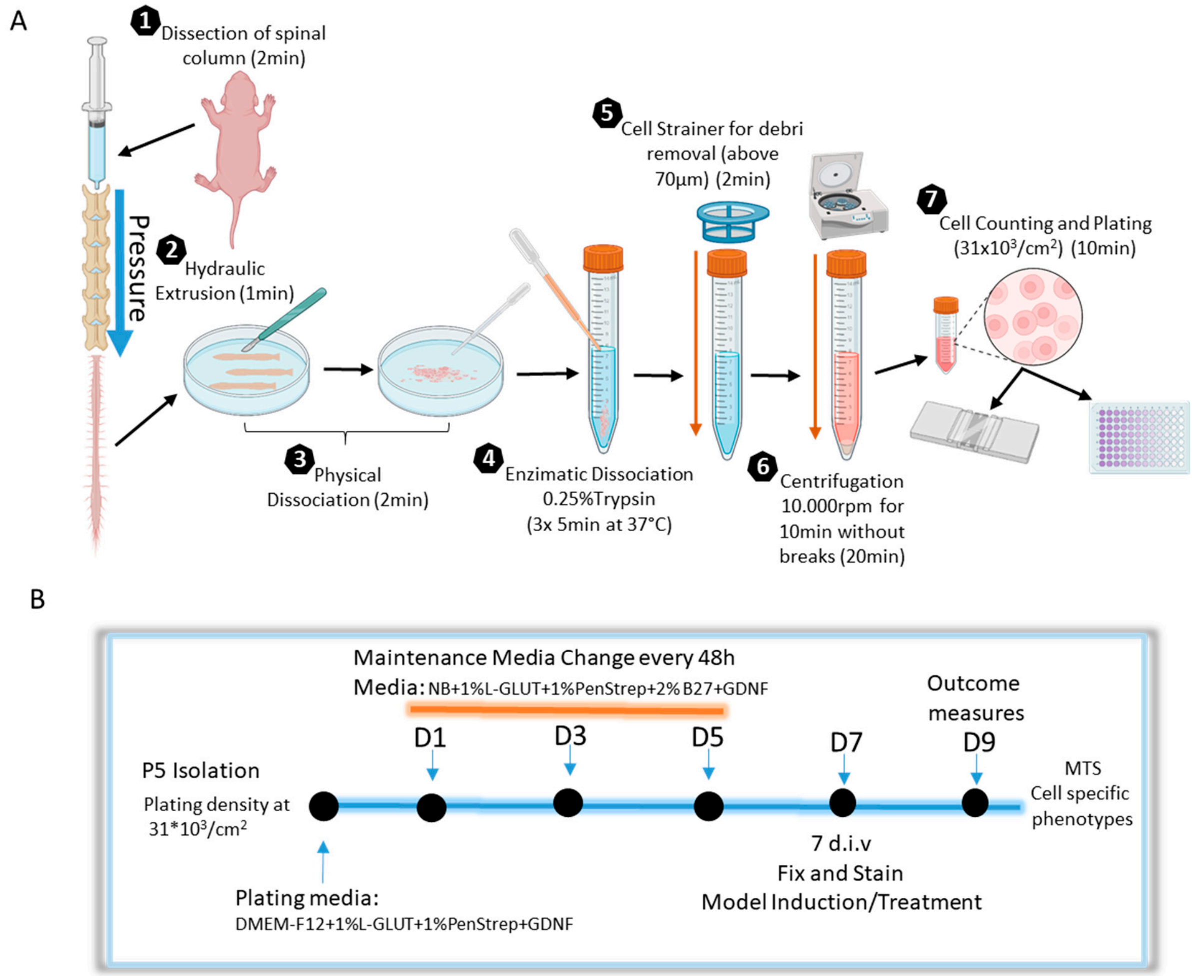

Supplementary Figure 1. Protocol for mixed spinal cord cells isolation and cell culture paradigm for sorbitol injury and treatments. (**A**) Step-by-step protocol for mixed spinal cord cell isolation with modifications from [1]. (**B**) Experimental and temporal cell culture paradigm for maintenance, injury induction and treatment of mixed spinal cord cells.

Supplementary Figure 2.

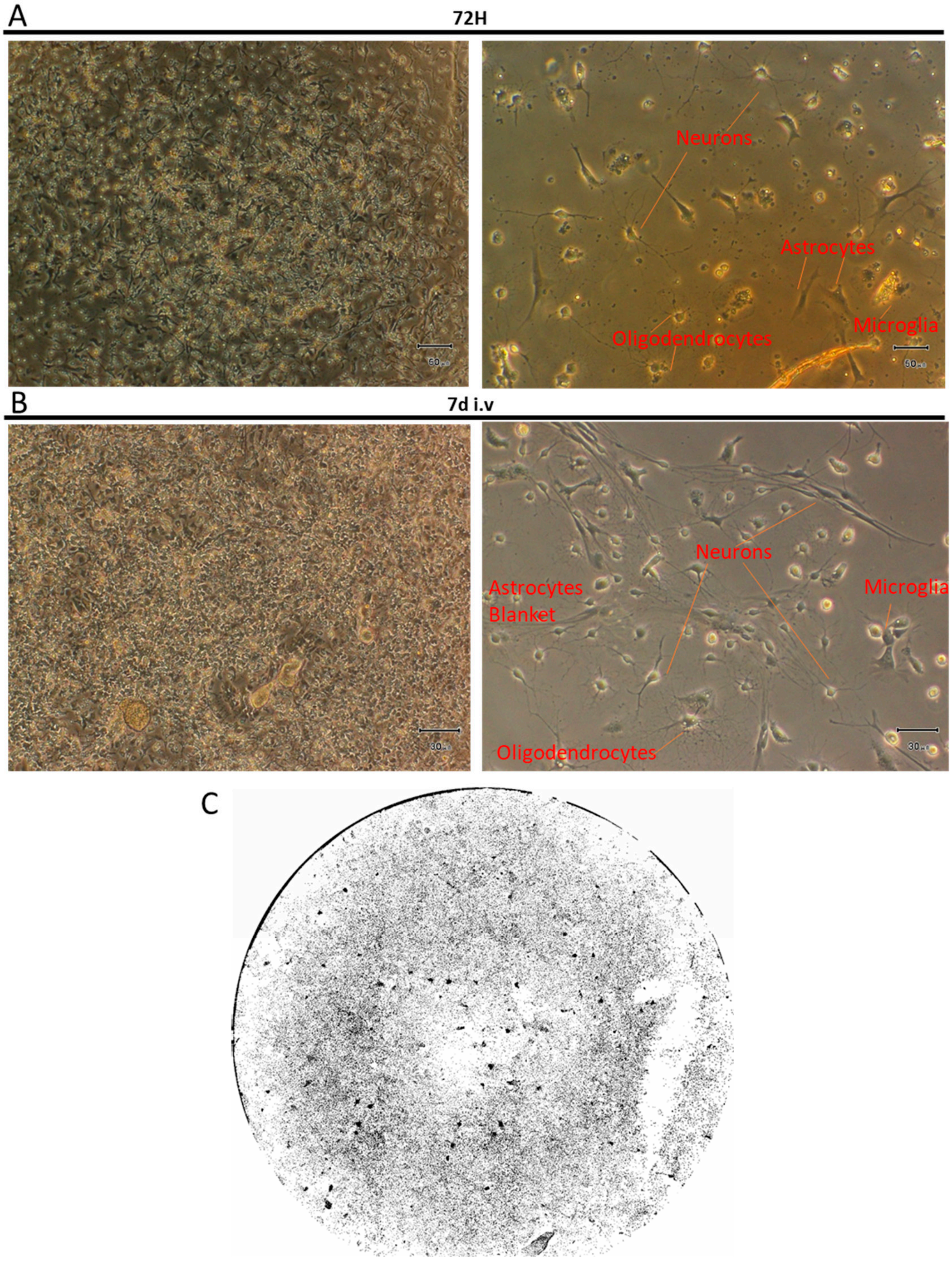

Supplementary Figure 2. Assessment of cellular adhesion and growth after plating. (**A**) Brighfield 4x and 10x (Right) showing adhered cells and different cell populations at 72-hours post plating. (**B**) Brighfield 4x and 10x (Right) maturation of different cell populations at 7-days of culture. (C) Cell nuclei segmentation representative of the whole-well DAPI quantification analysis.

Supplementary Figure 3.

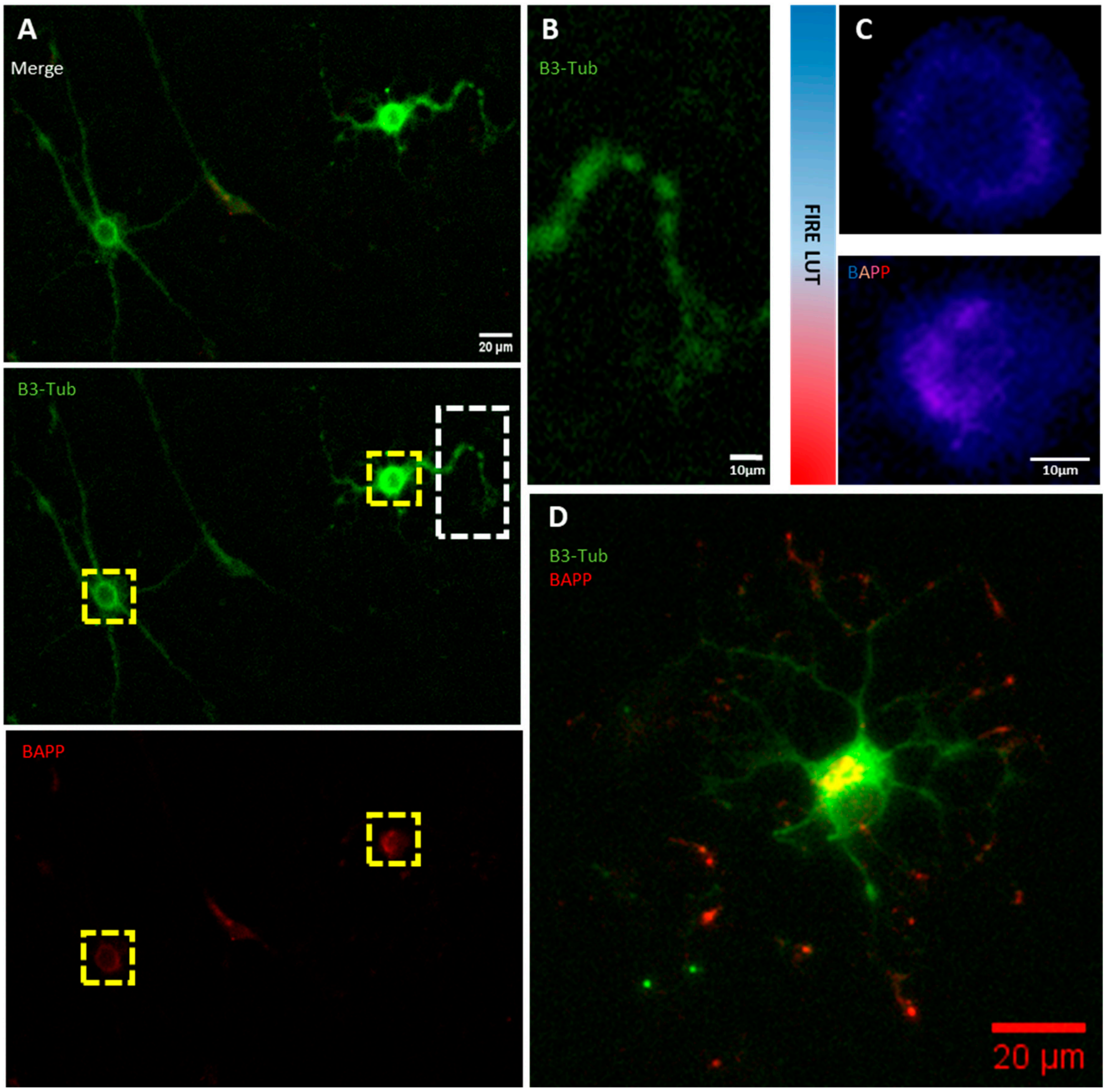

Supplementary Figure 3. Pipeline for assessment of neuronal phenotypic responses to hyper-tonic stress injury. Merged wide-field fluorescence images of B3+ neurons presenting different degrees of B-APP signal (left-considered negative) and (right-considered positive). (B) Axonal fragmentation associated with increased somatic expression of B-APP. (C) Changes in the expression pattern of B-APP in the soma of surviving neurons shown by a FIRE-LUT transformation of the raw image. (D) Emergence of somato-dendritic expression of B-APP after hyper-tonic stress injury.

Supplementary Figure 4.

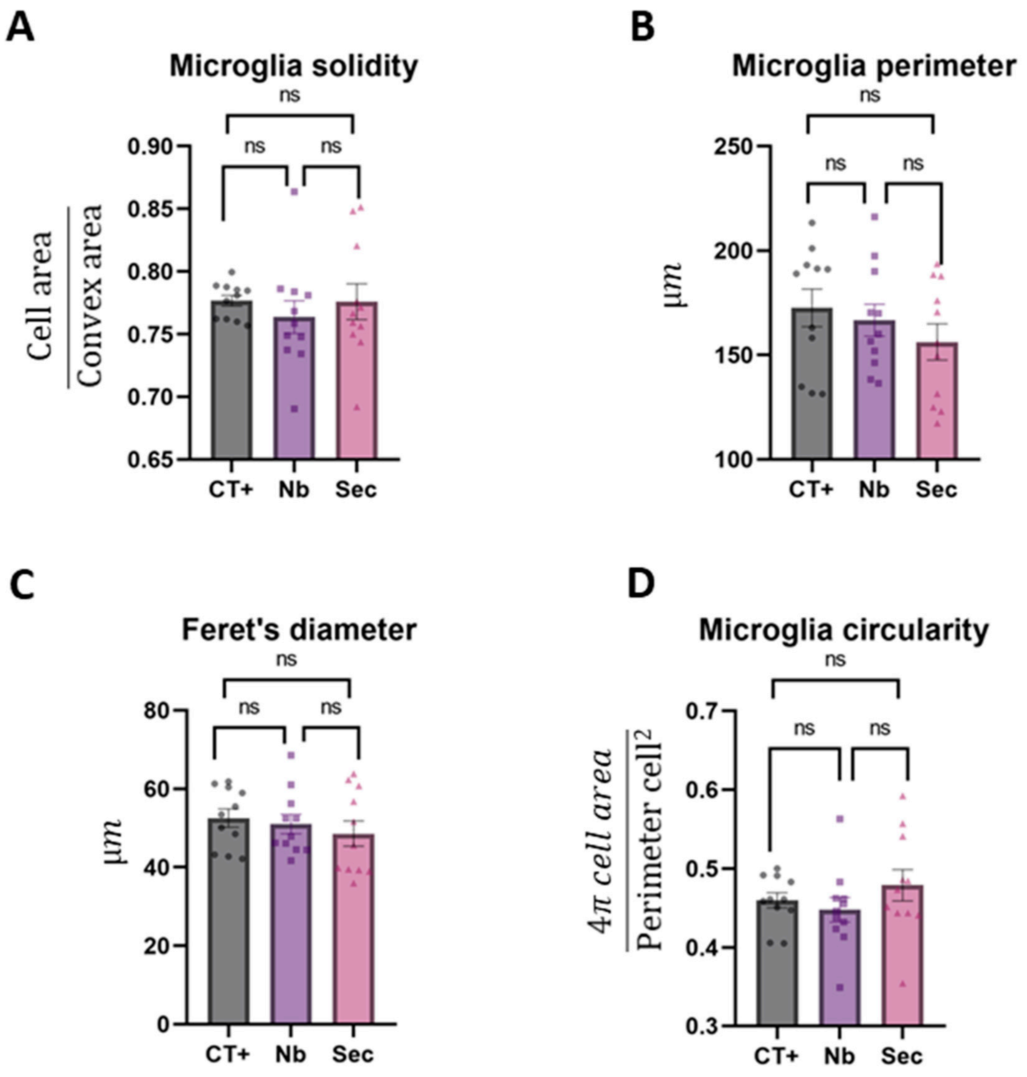

Supplementary Figure 4. Microglial morphologic responses to hyperosmotic stress injury and effects of the hASC secretome treatment. Microglial solidity (A). Microglial perimeter (B). Feret's diameter (C). Microglial circularity (D). Data shown as mean ± SEM. Individual data points in graphs represents biological samples (cell culture wells) and are representative of 3 independent assays. One-way ANOVA followed by Tukey post-hoc test. ns = non-significant.
